# A New Adjuvant MTOM Mediates *Mycobacterium tuberculosis* Subunit Vaccine to Enhance Th1-Type T Cell Immune Responses and IL-2^+^ T Cells

**DOI:** 10.3389/fimmu.2017.00585

**Published:** 2017-05-18

**Authors:** Qi Yu, Xiaochun Wang, Xionglin Fan

**Affiliations:** ^1^Department of Pathogen Biology, School of Basic Medicine, Tongji Medical College, Huazhong University of Science & Technology, Wuhan, China; ^2^School of Basic Medicine, Guiyang Traditional Chinese Medical College, Guiyang, China

**Keywords:** adjuvant, MTOM, *Mycobacterium tuberculosis*, Th1-type response, IL-2^+^ T cells

## Abstract

The only licensed vaccine *Mycobacterium bovis* Bacillus Calmette–Guérin (BCG) cannot prevent the prevalence of tuberculosis (TB), which remains a major public health problem worldwide. A more effective TB vaccine than BCG is urgently needed. Subunit vaccine is a promising strategy, and suitable adjuvants will benefit the development of effective TB subunit vaccines. MTO, consisting of monophosphoryl lipid A (MPLA), trehalose-6,6′-dibehenate (TDB), and MF59, was developed as an adjuvant of TB vaccine because of its ability to evoke the Th1-type T cell responses, while it is insufficient to induce single and multifunctional IL-2^+^ T cells and has a limited ability to confer protection against *Mycobacterium tuberculosis* infection. Heat-killed *Mycobacterium vaccae* (Mv), which can evoke cytotoxic CD8^+^ and CD4^+^ T cell responses and has adjuvanticity, was, in this study, combined with MTO to produce a new adjuvant, called MTOM. The TB fusion protein Rv3407-PhoY2-Ag85A-Rv2626c-RpfB (WH121) was mixed with MTO, Mv, and MTOM to produce three subunit vaccines, and the protective efficacy and immune responses were compared in C57BL/6 mice. WH121/MTOM provided better protection against TB than the other two vaccines, matching the performance of BCG vaccine. MTOM showed stronger ability to increase single and multifunctional IL-2^+^ T cells and induce Th1-type responses than MTO or Mv. Therefore, MTOM might be a promising adjuvant that could contribute to the development of TB subunit vaccines.

## Introduction

*Mycobacterium bovis* Bacillus Calmette–Guérin (BCG), an attenuated live strain derived from *M. bovis* and the only licensed vaccine for tuberculosis (TB), has been vaccinated in neonates worldwide with high coverage since the 1970s. However, TB remains the major threat among infectious diseases. There were approximately 10 million of new registered TB cases, and deaths from TB reached 1.4 million in 2015, respectively (1). Moreover, about one-third of the world’s population is estimated to be latent TB infections (LTBIs). Therefore, BCG cannot provide effective protection to control the prevalence of TB (2). A more effective TB vaccine than BCG is urgently needed.

CD4^+^ Th1 responses play a central role in the resistance to *Mycobacterium tuberculosis* (*M. tb*) infection (3, 4). Recent studies have suggested that CD4^+^ T cells are required to control TB progression and sustain multifunctional CD8^+^T cells during *M. tb* infection of non-human primates (5). Moreover, CD8^+^ T cells expressing Th1 cytokines probably contribute to the control of LTBI (6). Although Th17 responses are involved in immune protection against *M. tb* through recruiting and activating neutrophils at an early stage of infection, over-stimulation of Th17 responses leads to an exaggerated inflammation that instead contributes to tissue damage (7). Consequently, Th1-type T cell-mediated immunity is an attractive target for the development of new TB vaccines (8). Of different strategies, almost half of vaccine candidates in clinical trials belong to the type of subunit protein vaccination, which is generally accepted as a promising immunization strategy against *M. tb* (9). However, recombinant proteins are usually poorly immunogenic and the formulation in adjuvants is required to strengthen the magnitude of the responses to the protein antigen or to alter the type of immune responses induced (10). Alum adjuvant is widely used in human vaccines and mainly induces antibody response (11). Water-in-oil adjuvant MF59 (12) and AS03 (13) promote the generation of influenza-specific antibody. The adjuvant AS04, composed of monophosphoryl lipid A (MPLA) adsorbed on aluminum salt, is utilized in human papilloma virus and hepatitis B virus vaccine preparations to increase antigen-presenting cells (APCs) and the levels of IL-6 and TNF-α (14, 15). There are indeed other adjuvants in the pipeline that are known to induce Th1 immune response (16). CAF01, containing trehalose-6,6′-dibehenate (TDB), has been found to promote Th1 and Th17 immune responses in clinical Phase II trials (17). In our previous studies, we mixed MPLA and TDB in MF59 to produce a novel adjuvant, MTO. Although MTO-adjuvanted A1D4 subunit vaccine can elicit Th1-type immune response, its ability to induce single and multifunctional IL-2^+^ T cells is insufficient, which thus resulted in the inferior protective efficacy against *M. tb* infection to BCG (18). However, these adjuvants provide insufficient enhancement to the Thl-type T cell immune response and IL-2^+^ T cells. The appropriate, licensed adjuvants are required for the development of TB subunit vaccines.

Previously, Skinner et al. found that heat-killed *Mycobacterium vaccae* (Mv) had adjuvanticity and could evoke cytotoxic CD8^+^ and CD4^+^ T cells responses in immunized mice (19). In our earlier work, mouse immunization with live Mv strains induced protective immune responses against *M. tb* (20). In order to improve the adjuvant effect of MTO, we first incorporated heat-killed Mv in MTO to construct a new adjuvant, MTOM. A polyprotein Rv3407-PhoY2-Ag85A-Rv2626c-RpfB, which was based on five immunodominant antigens expressed during different stages of TB infection, WH121 for short, that had previously been shown in mice to be a promising subunit vaccine candidate (21), was mixed with the adjutants MTO, Mv, and MTOM, and the immune responses and the protective efficacy on mice exposed to *M. tb* were observed and compared.

## Results

### MTOM-Adjuvanted WH121 Subunit Vaccine Affords Improved Protective Efficacy

**Figure 1:**
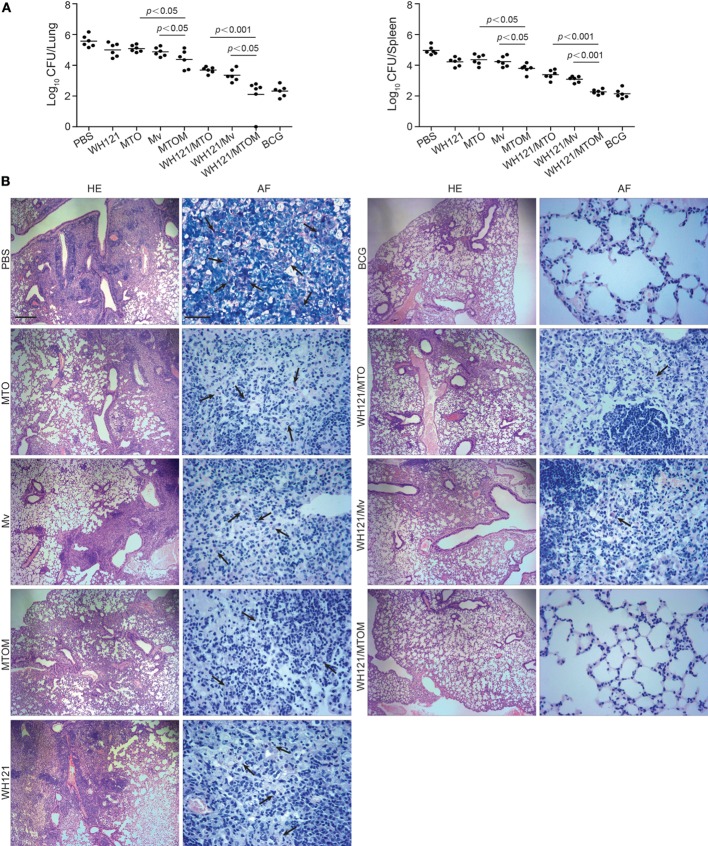
**Comparison of protective efficacy of different vaccines**. **(A)** Six weeks after final immunizations, C57BL/6 mice (*n* = 6) were intranasally challenged with about 100 CFU virulent *M. tb* H37Rv strain. Four weeks after challenge, the bacterial load within lungs and spleens of mice in different groups was assessed and represented as mean (±SEM) log_10_ CFU/organ (*n* = 6). **(B)** Lung tissue was sectioned and stained with hematoxylin and eosin (HE) (scale bar, 400 µm) and acid-fast (AF) staining (scale bar, 50 µm). Arrows indicate AF-positive bacteria.

To compare the protection of WH121 protein combined with different adjuvants, C57BL/6 mice were challenged intranasally with live *M. tb* strain H37Rv after the last immunization. Protective efficacy was assessed by comparison of the bacterial load in lung and spleen and by the lung pathology at 1 month post infection. As shown in Figure 1A, the highest bacterial load in both lung and spleen was observed in the PBS control group. Either adjuvant MTO, Mv, or WH121 alone also slightly inhibited the growth of *M. tb* in both organs, but the effects were inferior to those offered by MTOM (*P* < 0.05). C57BL/6 mice vaccinated with WH121 subunit vaccines showed significantly lower colony-forming unit (CFU) values in the lung and spleen than mice treated with adjuvant alone. Importantly, the bacterial load in WH121/MTOM group was lower than those in WH121/MTO and WH121/Mv groups, and similar to the results of the BCG group.

As shown in Figure 1B, mice in the PBS control group presented the most severe histopathology with extensive fibrosis, perivasculitis, pulmonary alveolitis, and lymphocyte infiltrates, and acid-fast (AF)-positive bacilli were found throughout the whole lung section. Mice in the adjuvant or WH121 protein alone groups also showed dispersed AF-positive bacilli in the lung and exhibited similar, less severe pathological changes to those observed in the PBS control mice. In contrast, the pulmonary lesions and inflammation of mice in the WH121/MTO and WH121/Mv groups were substantially reduced and a few AF-positive bacilli were detected in the alveolar tissue. Remarkably, the mice vaccinated with WH121/MTOM exhibited less pronounced pathological manifestation than those vaccinated with WH121/MTO and WH121/Mv. They were comparable to the effects observed in BCG-immunized mice (Figure 1B). All these results demonstrated that MTOM-adjuvanted WH121 performed better than the combinations of MTO or Mv and WH121, and its protective efficacy matched with that of BCG.

### WH121/MTOM Subunit Vaccine Induced Stronger Antibody Responses

To determine the levels of antibody induced by different WH121 vaccines, mice were vaccinated twice, 3 weeks apart with WH121/MTO, WH121/Mv, or WH121/MTOM. Six weeks after the final vaccination, WH121-specific antibodies, including IgG, IgG1, and IgG2a, were titrated by ELISA.

**Figure 2:**
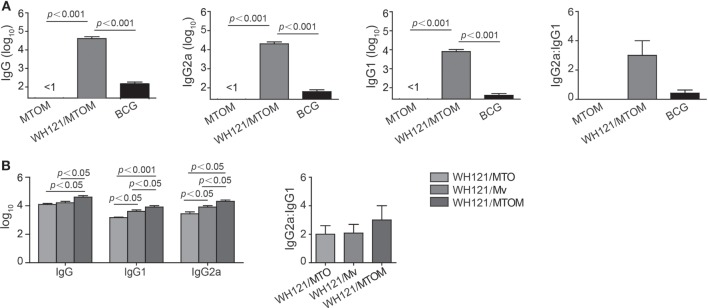
**Effect of MTOM-adjuvanted WH121 protein on serum antibody levels in immunized mice**. Six weeks after final immunization, the levels of serum IgG, IgG1, and IgG2c (replaced with IgG2a) antibodies from immunized mice were detected using ELISA. **(A)** WH121/MTOM induced IgG, IgG1, and IgG2a antibodies specific to WH121. **(B)** Comparison of antibody levels induced by WH121/MTO, WH121/Mv, and WH121/MTOM. Results are shown as mean (±SEM) log_10_ end point titers and the ratio of IgG2a:IgG1 in the differently vaccinated groups (*n* = 3).

As shown in Figure 2, there was no WH121-specific antibody response to adjuvant MTO, Mv, or MTOM alone. Compared to BCG, mice vaccinated with WH121/MTOM produced higher levels of WH121-specific IgG, IgG1, and IgG2a (*P* < 0.001). The ratio of IgG2a/IgG1 substantially increased (Figure 2A), which indicated that WH121/MTOM elicited a shift of the IgG subclass toward a Th1-type response. Furthermore, WH121/MTOM induced higher levels of these antibodies than WH121/MTO or WH121/Mv (Figure 2B), and the conversion trend of the IgG subclass toward Th1-type responses was more obvious (Figure 2B).

### Th1 Cytokines in Response to Subunit WH121/MTOM

**Figure 3:**
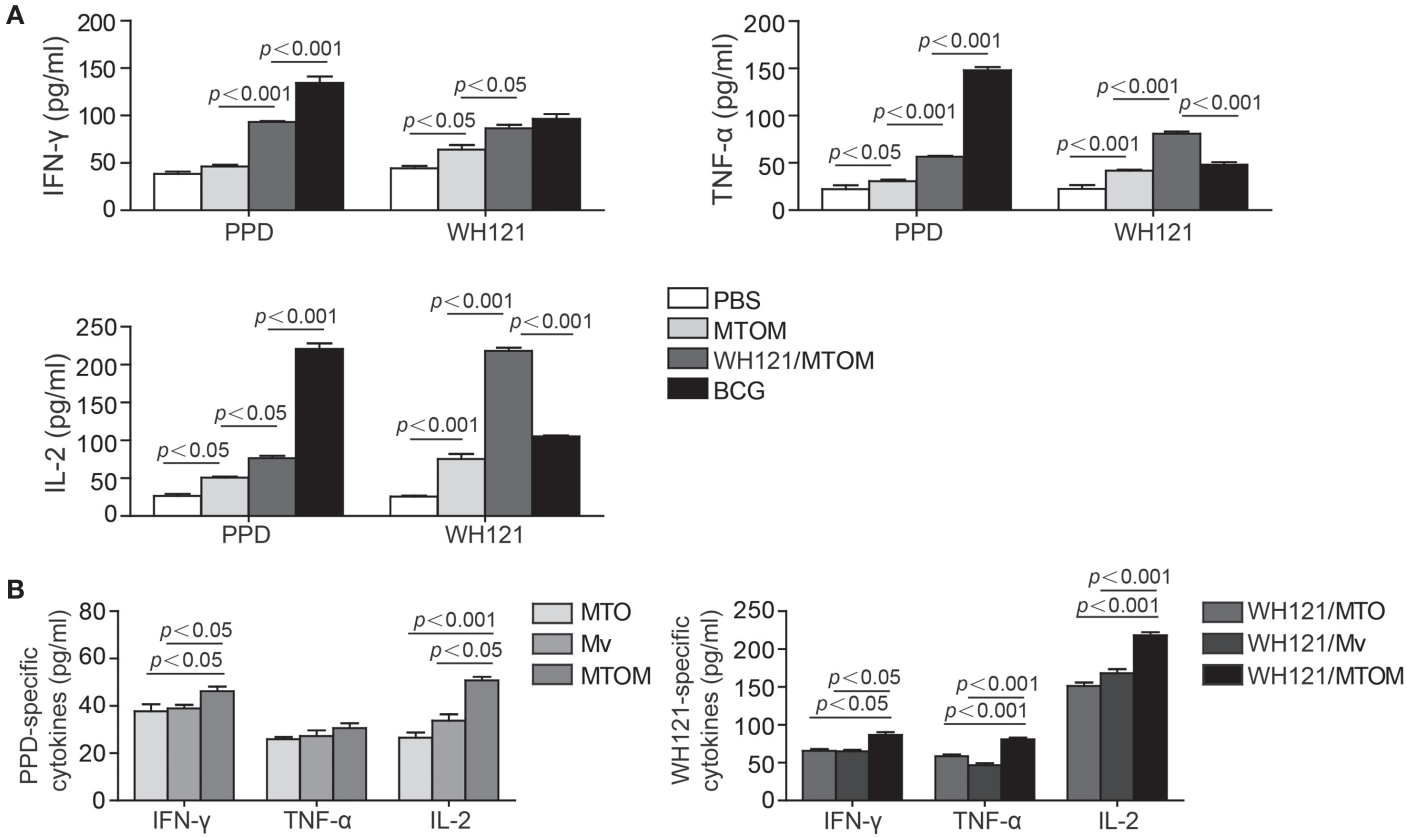
**The levels of IFN-γ, TNF-α, and IL-2 secreted by splenocytes from different groups**. Six weeks after the last immunization, splenocytes were collected from each mouse (*n* = 3). A total of 5 × 10^6^ cells were added to each well of 24-well microtiter plates and incubated with WH121 protein (10 µg), PPD (10 µg), or complete RPMI1640 medium for 24 h (for IL-2 detection) or 72 h (for IFN-γ and TNF-α detection) at 37°C in 5% CO_2_. The cytokine concentrations in the suspension were determined by ELISA. **(A)** PPD- or WH121-specific cytokines induced by Bacillus Calmette–Guérin (BCG), PBS, WHI121/MTOM, and MTOM. **(B)** PPD-specific cytokines induced by MTO, *Mycobacterium vaccae* (Mv) and MTOM, and WH121-specific cytokines induced by WH121/MTO, WH121/Mv, and WH121/MTOM. The results are shown as mean ± SD (pg/mL).

In this study, IFN-γ, TNF-α, and IL-2 released by Th1 cells were detected to explore the effects of different adjuvant WH121 formulations on Th1-type immune responses. The results showed the splenocytes in the PBS control group secreted very low levels of IFN-γ, TNF-α, and IL-2 in response to PPD or WH121 (Figure 3A). The levels of all antigen-specific Th1 cytokines except PPD-specific IFN-γ were significantly higher in the MTOM alone group than in the PBS control group (Figure 3A). The WH121/MTOM vaccine induced higher levels of cytokine responses to PPD and WH121 than MTOM did (*P* < 0.05, *P* < 0.001, Figure 3A). Among all groups, the highest levels of PPD-specific Th1 cytokines were produced in the BCG group. However, mice vaccinated with WH121/MTOM produced higher levels of WH121-specific TNF-α and IL-2 than BCG did (*P* < 0.001, Figure 3A). In addition, MTOM adjuvant also stimulated higher levels of PPD-specific IFN-γ and IL-2 than did MTO or Mv (*P* < 0.05, *P* < 0.001, Figure 3B). The levels of WH121-specific cytokines induced by WH121/MTOM were higher than those induced by WH121/MTO or WH121/Mv (*P* < 0.05, *P* < 0.001, Figure 3B). These results demonstrated that the adjuvant MTOM could promote the secretion of Th1 cytokines to a remarkable extent. The protection induced by MTOM formulated in the subunit vaccine was in a large part dependent on Th1-type responses.

### Different Adjuvants Induced Different Multifunctional T Cell Subsets

**Figure 4:**
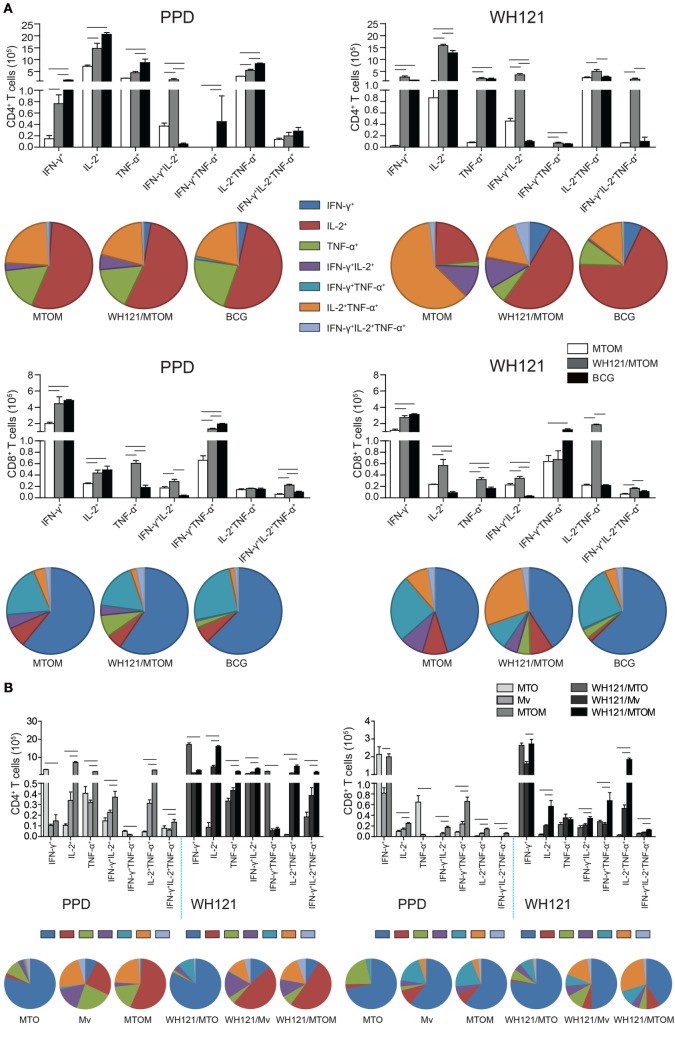
**PPD- and WH121-specific multifunctional CD4^+^ and CD8^+^ T cell responses**. Splenocytes from each mouse in each group were obtained and counted. A total of 5 × 10^6^ cells per well were added to 24-well plates and stimulated with WH121 protein (10 µg) or PPD (10 µg). Intracellular cytokine profiles for IFN-γ, TNF-α, and IL-2 in individual cells were detected using multicolor flow cytometry by gating for CD4^+^ or CD8^+^ T cells. **(A)** The numbers of multifunctional CD4^+^ and CD8^+^ T cells induced by WH121/MTOM. **(B)** PPD-specific CD4^+^ and CD8^+^ T cells elicited by MTO, *Mycobacterium vaccae* (Mv), and MTOM and WH121-specific CD4^+^ and CD8^+^ T cells induced by WH121/MTO, WH121/Mv, and WH121/MTOM. The number of cells in the PBS group, considered a background value, was subtracted. Each possible combination of cytokines is shown on the *x*-axis of the bar chart, and the absolute number of antigen-specific T cells expressing any combination of cytokines is shown as mean ± SEM in the different groups (*n* = 3). ^-^ represents *P* < 0.05.

To assess the effects of WH121 protein formulated with different adjuvants on multifunctional T cell subsets, biomarkers from splenocytes of vaccinated mice were stained and analyzed by multi-parameter flow cytometry. The absolute number of PPD-specific single IFN-γ^+^, single IL-2^+^, single TNF-α^+^, IFN-γ^+^TNF-α^+^, and IL-2^+^ TNF-α^+^ CD4^+^ T cells was significantly higher in the BCG group than in the MTOM alone group (*P* < 0.05, Figure 4A). All the combinations of CD4^+^ T cell cytokine responses to WH121 were elevated to a greater degree in the WH121/MTOM group than in the MTOM alone group (*P* < 0.05, Figure 4A). In particular, WH121/MTOM showed higher levels of both PPD- and WH121-specific IFN-γ^+^IL-2^+^ CD4^+^ T cells than BCG (*P* < 0.05, Figure 4A). In addition, relative to MTOM, the BCG vaccine induced mostly PPD-specific single IFN-γ^+^ and IFN-γ^+^TNF-α^+^ CD8^+^ T cells, and the following cells: single IL-2^+^, single TNF-α^+^, and IFN-γ^+^TNF-α^+^IL-2^+^ CD8^+^ T cells; while the WH121/MTOM vaccine increased six combinations of CD8^+^ T cell responses to WH121 in addition to IFN-γ^+^TNF-α^+^ CD8^+^ T cells (*P* < 0.05, Figure 4A). Notably, the frequency with which WH121-specific CD8^+^ T cells expressed single, double, or triple IL-2^+^ was higher in the WH121/MTOM group than in the BCG group (*P* < 0.05).

As shown in Figure 4B, MTO elicited mainly single IFN-γ^+^ and single TNF-α^+^ CD4^+^ and CD8^+^ T cells, which is consistent with previous findings (18). Mv induced mainly single IL-2^+^ CD4^+^ T cells and single IFN-γ^+^ CD8^+^ T cells. Interestingly, MTOM elicited significantly more single, double, and triple IL-2^+^, CD4^+^, and CD8^+^ T cells than MTO or Mv (*P* < 0.05, Figure 4B). The multifunctional T cell responses induced by three WH121 subunit vaccines were essentially the same as their corresponding adjuvant alone. WH121/MTOM-vaccinated mice showed greater proportions of cells expressing IL-2 (either alone or co-expressed with other cytokines) than those vaccinated with WH121/MTO or WH121/Mv (Figure 4B).

## Discussion

Adjuvant plays a pivotal role in the development of new TB subunit vaccine candidates. We combined heat-killed Mv with MTO to develop a next-generation adjuvant, MTOM. Combined with a fusion protein WH121, it helped to evoke more robust antigen-specific Th1-type immune responses and generate more IL-2-positive multifunctional T cells than did MTO or Mv, thus providing the same protective efficacy against TB as the BCG vaccine. These indicate that the adjuvant MTOM is an efficient Th1-type immunostimulator and can produce TB vaccines with significantly improved immune effects.

*M. tb* is infected naturally through respiratory route and engulfed by alveolar macrophages and dendritic cells (DCs). Pattern recognition receptors (PRRs) on the cell surface of APCs, including toll-like receptors (TLRs) and C-type lectin Mincle receptor, can recognize *M. tb* and its components, which will lead to the activation of innate immunity and the initiation of adaptive immunity against *M. tb* infection (22). We assumed that an effective subunit vaccine for TB might be developed based on the combination of different agonists of PRRs and multistage antigens of *M. tb*. The MF59 adjuvant is reported to target DCs, monocytes/macrophages, and granulocytes and to promote antigen presentation, immune cell differentiation, and the release of cytokines (23). MPLA is the first well-defined TLR4 agonist to induce robust CD4^+^ T cell responses and the production of type I interferons through TRIF and MyD88 signaling pathways (24, 25). The immunostimulation of TDB as the agonist of Mincle triggers the FcRγ-Syk-Card9 pathway in APCs, and then facilitates the activation of APCs and T cells (26). Fochesato et al. reported that MPLA is a major component of AS01 adjuvant, which has been used with varicella-zoster virus vaccine to increase the number of CD4^+^ T cells expressing IFN-γ (27). Derrick et al. found the combinations of TDB and DDA boosted BCG to elevate the frequencies of CD4^+^ and CD8^+^ T cells expressing IFN-γ and IFN-γ/TNF-α (28). As expected, both MTO and MTOM containing MF59, MPLA, and TDB were able to increase the levels of IgG2a- and Th1-dominant cytokines and increase the number of multifunctional CD4^+^ and CD8^+^ T cells.

*Mv* is a non-pathogenic mycobacterium, which is the only immunoregulatory agent for the treatment of TB recommended by WHO (29). It is characterized by significant safety and efficacy (30). Unlike the materials used to produce the BCG vaccine, Mv is used as a heat-killed organism, so it provides less protection, and it may be more effective as an immunotherapy agent than as a vaccine agent. Mv has immunoregulatory components such as oligonucleotides (ODN) containing unmethylated CpG motifs that can be recognized by TLR9 and so enhance a Th1-biased immune response and CD8^+^ T cell-mediated response (31, 32). Mv might express some of the same antigens as *M. tb* (33), which together are processed and cross-presented on MHC class I molecules on the APC, and so lead to the production of specific CD8^+^ cytotoxic T cells. Recently, *in vitro* and *in vivo* studies have also confirmed that Mv can downregulate Th2 and enhance Th1 responses through the induction of regulatory T cells and DCs (34).

In this study, we used multi-parameter flow cytometry to assess the magnitude and quality of the T cell responses in immunized mice. We found that Mv could increase the number of IL-2^+^ T cells to a certain extent, and adding it into MTO triggered multifunctional T cell responses quite different from those induced by MTO or Mv alone. Correspondingly, the antigen-specific CD4^+^ and CD8^+^ T cells induced by WH121/MTO, WH121/Mv, and WH121/MTOM also presented phenotypic and functional heterogeneity. According to these results, the levels of single, double, and triple IL-2^+^, CD4^+^, and CD8^+^ T cells from MTOM-immunized mice were significantly higher than in the MTO and Mv groups. Likewise, mice vaccinated with WH121/MTOM showed many more T cells expressing IL-2 than WH121/MTO- or WH121/Mv-treated mice. Although it is unclear whether the increase in IL-2 synthesis is correlated with greater vaccine effectiveness, elevated levels of IL-2 have been shown to contribute to the maintenance of cellular immunity and granuloma formation (35) and to augment effective immune responses via expansion of activated CD8^+^ T cells. They can also induce the expression of cytokines (e.g., IFN-γ) and the generation of immune memory CD8^+^ T cells (36, 37). The expression of IL-2 has been found to be associated with the successful long-term survival of activated CD4^+^ T cells *in vivo* (38). WH121/MTOM vaccine significantly increased the number of CD4^+^ or CD8^+^ T cells co-expressing multiple cytokines. Recent reports from various pathogens in different animal models have shown that multifunctional T cells that express IFN-γ^+^, TNF-α^+^, and IL-2^+^ are functionally superior to their single positive counterparts (39, 40). The concomitant production of the high levels of cytokines contributes to successful elimination of intracellular pathogens (39, 41). Aagaard et al. demonstrated that the H56/CAF01 vaccine improved the multifunctional CD4^+^ T cell populations secreting IFN-γ^+^IL-2^+^ and IFN-γ^+^IL-2^+^TNF-α^+^ and evoked a level of protection against TB comparable to that evoked by BCG (42). In our previous study (21), WH121/DMT elicited the higher numbers of IFN-γ^+^IL-2^+^ CD4^+^ T cells than did BCG. The same investigation of WH121 in the adjuvant of MTOM-vaccinated mice also showed pronounced induction of WH121-specific IFN-γ^+^ IL-2^+^ and triple-positive CD4^+^ T cells. Collectively, Mv improved the immunomodulatory effects of adjuvant MTO. The enhancement of anti-TB protection induced by WH121/MTOM is probably partially attributable to changes in multifunctional T cell responses. Specifically, the MTOM adjuvant promotes the generation and maintenance of Th1-type responses and IL-2^+^ T cells.

Because each component of the fusion protein, WH121, was not reported to function as adjuvant (10), WH121 protein-alone-treated mice only showed slight protection against *M. tb* infection. Differential immunogenicity between different adjuvants alone, or between different adjuvanted WH121 vaccines, was analyzed in this study as previously performed (43, 44). All of these confirm that the synergistic action of both adjuvant and protein plays important roles in the efficacy of TB subunit vaccines. Differing from other studies (43, 44), BCG provided two to three log protections compared to PBS control in our experiments. Multiple factors such as different BCG substrain itself, immunization period, and infection time, dose and route might influence the results, as described earlier (18, 21). Moreover, the virulence of *M. tb* H37Rv strain is highly variable and laboratory dependent (43, 44). In the mice immunized with WH121/MTOM, Th1 cytokine response was better compared to that induced by BCG immunization. However, the protective efficacy of WH121/MTOM was not significantly higher than BCG. Although Th1 responses are believed to be the most important protective mechanism against *M. tb*, the role of innate immunity also cannot be ignored (45). As a live bacterium, the protective mechanism of BCG has remarkable difference from that of subunit vaccine. As a result, more research is needed to further enhance the immunogenicity of our subunit protein vaccine, and to optimize dose administration.

In conclusion, this is the first report to combine Mv to MTO to form a new water-in-oil adjuvant, called MTOM. This adjuvant is found to augment Th1-type responses and IL-2^+^ T cells, thus could be used for the development of vaccine candidates against other intracellular pathogens. Our studies also demonstrate that MTOM-based WH121 vaccine has better immunogenicity and protection than other adjuvanted vaccines and is comparable to BCG in effectiveness. This work may help to accelerate the development of TB vaccines, and it lays the foundation for further preclinical evaluation of WH121/MTOM in other animal models.

## Materials and Methods

### Preparation of Adjuvants and Vaccines

The WH121 fusion protein expressed by a genetically engineered expression system in *E. coli* was constructed and prepared in our laboratory. Water-in-oil adjuvant, MTO, was prepared as described previously (18). Mv was cultivated in sterile medium (2.5 g/L yeast extract, 5 g/L tryptone, 1 g/L glucose) at 37°C. The mycobacteria were harvested by centrifugation and transferred to sterile 7H9 medium (Difco, USA, Cat. no. 0189627) with 10% ADC and 0.5% glycerol at 37°C for 24 h. Culture supernatant was discarded after centrifugation. Then, the precipitate was concentrated and weighed. Heat-killed Mv was prepared from the mycobacterial pellet resuspended in PBS at 10 mg/mL (equivalent to 10^10^ CFU/mL) and autoclaved for 15 min at 121°C. The lysate of Mv was diluted to 5 mg/mL (19). MTOM adjuvant was prepared as an evenly emulsified mixture of 1 mL MTO and 1 mL Mv lysate solution. Finally, 200 µL of immunization dose of each WH121 subunit vaccines was prepared by mixing 20 µg WH121 protein and 50 µL MTO or Mv or 100 µL MTOM in 10 mM Tris buffer, respectively.

### Mice and Immunizations

C57BL/6 female mice aged 6–8 weeks were obtained from the Center for Animal Experiments of Wuhan University (Wuhan, China) and maintained under specific pathogen-free conditions in a BSL-3 laboratory. For this study, 200 µL of WH121 vaccine preparations was given to mice subcutaneously (s.c.) twice at 3-week intervals. WH121 protein and adjuvants, MTO, Mv, and MTOM, were similarly administered for comparison. The BCG China vaccine was used as a positive control, which was administered once (1 × 10^6^ CFU) s.c. at the time of the first vaccination. PBS served as a negative control.

### Infection with *M. tb* H37Rv and Evaluation of Vaccine-induced Immune Protection

*M. tb* H37Rv was grown to mid-log phase in sterile Middlebrook 7H11 agar (Difco, USA, Cat. no. 4364531) with 10% ADC and 0.5% glycerol, and harvested and weighed. Then, the bacterial suspension was prepared in PBS at 0.1 mg/mL (about equivalent to 10^6^ CFU/mL) by a sterile glass grinder. Six weeks after their last immunization, mice in each group were first anesthetized by intraperitoneal injection with 120 µL of 0.8% pentobarbital sodium, namely 0.96 mg/15 g weight of mice. 20 µL of the bacterial suspension in a microinjector was then used to infect each mouse intranasally (18, 21, 46, 47). After infection, three mice in the PBS control group were killed on the next day, and the actual infection dose was determined by enumeration of lung bacterial load. Four weeks later, the mice were sacrificed for the evaluation of protective efficacy. Lungs and spleens were removed aseptically and homogenized in sterile 0.05% PBS-Tween80, then plated at 10-fold serial dilutions on Middlebrook 7H11 agar plates. 2-thiophenecarboxylic acid hydrazide (2 μg/mL) was supplemented to plates to inhibit the possible growth of residual BCG in the BCG group. Plates were cultured at 37°C for 4 weeks, and the bacterial load was shown as mean (log_10_ CFU) ± SEM per organ for each group (*n* = 6). Right lung lobes from the differently vaccinated groups (*n* = 3) were fixed in 10% buffered formaldehyde and embedded in paraffin. Slices (5 µm thick) were cut and stained with hematoxylin and eosin (HE) or AF staining. Pathological changes were analyzed with light microscopy (Nikon ECLIPSE E100, Japan).

### Detection of WH121-Specific Antibody Levels

Six weeks after the last immunization, sera were collected from vaccinated mice. WH121-specific end point titers for IgG1, IgG2c, and total IgG antibodies were detected by ELISA. Using previously described methods (48), microtiter plates were pre-coated with 100 µL WH121 protein (5 µg/mL) in carbonate/bicarbonate buffer (pH 9.6) overnight at 4°C and blocked with 1% BSA in PBST for 1 h at 37°C. Horseradish peroxidase-conjugated rabbit anti-mouse IgG (1/5,000; Abcam, UK, Cat. no. Ab6789), IgG1 (1/10,000; Abcam, UK, Cat. no. Ab97240), or IgG2c (1/10,000; Abcam, UK, Cat. no. Ab97255) were added to the plates. 100 µL 3,3′5,5′-tetramethylbenzidine substrate was used for visualization at 37°C. Plates were read at 450 nm using a Multiskan ELISA reader (Bio-tek, USA). Antibody titers in each group were determined by comparison to the PBS control group. The results are expressed as mean (±SEM) log_10_ end point titers, and as the ratio of IgG2c/IgG1.

### Antigen-Specific IFN-γ, IL-2, and TNF-α ELISA

Spleens were removed aseptically from immunized mice. Splenocytes were counted and plated at 5 × 10^6^ in each well of 24-well microtiter plate for 24 h (for IL-2 determination) and 72 h (for IFN-γ and TNF-α detection) at 37°C with WH121 (10 µg/mL). RPMI1640 medium served as a negative control; PPD (10 µg/mL; Statens Serum Institut, Denmark) served as a positive control. Supernatants were collected from three separate wells to assess the levels of IFN-γ, IL-2, and TNF-α using mouse IFN-γ (EK2802), IL-2(EK2025), and TNF-α ELISA kits (EK2822, all from Multi Sciences Ltd., Hangzhou, China) according to the manufacturer’s instructions. The results in this study are expressed as mean ± SD (pg/mL) of each group (*n* = 3).

### Detection of Multifunctional T Cells with Flow Cytometry

Splenocytes (5 × 10^6^) from three individual mice per group were prepared as mentioned earlier and plated in a 24-well plate, stimulated with WH121 (10 µg/mL) for 16 h, and incubated for another 4–6 h after the addition of 3 µg/mL brefeldin A and 2 µM monensin solution (eBioscience, USA). RPMI1640 medium provided a negative control, and PPD (10 µg/mL) was used as positive control. A cell stimulation cocktail (1 µg/mL; eBioscience, USA) was used to monitor cell responses. After washing in FACS buffer (1% FCS-PBS), the surface markers of cells were stained using anti-CD3-FITC (eBioscience, USA, Cat. no. 11-0032), anti-CD4-APC-cy7 (BD Biosciences, USA, Cat. no. BD-557871), and anti-CD8a PE (eBioscience, USA, Cat. no. 12-0081) mAbs at 4°C for 30 min in the dark. Cells were washed again and permeabilized using an Intracellular Fixation and Permeabilization Buffer Kit (eBioscience, USA). Anti-IFN-γ PerCP-Cyanine5.5 (eBioscience, USA, Cat. no. 45-7311), anti-TNF-PE-Cy™7 (eBioscience, USA, Cat. no. 25-7321), and anti-IL-2 APC (eBioscience, USA, Cat. no. 17-7021) mAbs were used for intracellular staining. The absolute number of CD4^+^ and CD8^+^ T cell subsets secreting single, double, or triple cytokines was determined using the LSRII multicolor flow cytometer (BD Biosciences, USA) and analyzed using FlowJo software. To provide baseline normal values, the number of cells from three mice in the PBS group was determined. The results are shown as mean ± SEM per group (*n* = 3).

### Statistical Analysis

Graph Pad Prism 5 software was used to analyze the data. Statistical analysis was performed using SPSS 18.0 software. One-way ANOVA or *t*-test was used to assess the difference between the different groups. *P*-values < 0.05 were considered significant.

## Ethics Statement

The experiments described in this study were performed in accordance with the guidelines of the Chinese Council on Animal Care. The research protocols were approved by the Committee on the Ethics of Animal Experiments of Huazhong University of Science and Technology.

## Author Contributions

XF designed this study. XW performed preceding experiments and assisted in data collection. QY conducted the experiments, analyzed the data, and prepared the manuscript. XF and QY revised the manuscript.

## Conflict of Interest Statement

The authors declare that the research was conducted in the absence of any commercial or financial relationships that could be construed as a potential conflict of interest.
